# Impact of prenatal ultrasound diagnosis for fetal renal abnormalities: Study Protocol

**DOI:** 10.1097/MD.0000000000017907

**Published:** 2019-11-11

**Authors:** Ying Chen, Wei Liu

**Affiliations:** a Department of Ultrosound, The First Hospital of Yulin; b Department of Ultrosound, Xing Yuan Hospital of Yulin, Yulin, China.

**Keywords:** fetal renal abnormalities, prenatal ultrasound diagnosis, sensitivity, specificity

## Abstract

**Background:**

This study will evaluate diagnostic accuracy and management outcome studies involving patients assessed with prenatal ultrasound diagnosis (PUD) for fetal renal abnormalities (FRA).

**Methods:**

We will search the following electronic databases of MEDLINE, EMBASE, Cochrane Library, Web of Science, Springer, Cumulative Index to Nursing and Allied Health Literature, Allied and Complementary Medicine Database, Chinese Biomedical Literature Database, and China National Knowledge Infrastructure for diagnostic accuracy studies from inceptions to the present without language restrictions. Two authors will independently screen studies, collect data, and assess methodological quality.

We will use RevMan V.5.3 and Stata V.12.0 software for data pooling and statistical analysis.

**Results:**

In this study, we will assess sensitivity, specificity, positive likelihood ratio, negative likelihood ratio, and diagnostic odds ratio to determine the diagnostic accuracy of PUD for the treatment of patients with FRA.

**Conclusion:**

This study will provide latest evidence for the diagnostic accuracy of PUD for FRA.

**Systematic review registration:**

PROSPERO CRD42019151306.

## 1 Introduction

Evaluation of fetal anatomy during the trimester period is very important and necessary for fetal.^[1–3]^ It has become a standard practice in most antenatal care set-ups, and has helped to diagnose most structural abnormalities in the fetus.^[4–7]^ It has been estimated that fetal renal anomalies (FRA) accounts for about 20% of all congenital abnormalities in fetal.^[8–11]^ Moreover, this condition can significantly affect prenatal morbidity and mortality.^[12–13]^ Fortunately, prenatal ultrasound diagnosis (PUD) has been reported to identify such condition accurately.^[14–20]^ However, no study has systematically explored this issue. Thus, this study will investigate the diagnostic accuracy of PUD for FRA systematically.

## 2 Methods

### 2.1 Objective

This study aims to review clinical outcomes of patients investigated the diagnostic accuracy of PUD for FRA.

### 2.2 Inclusion criteria for study selection

#### 2.2.1 Type of studies

This study will consider case-controlled studies reporting the diagnostic accuracy of PUD for FRA for inclusion. However, we will exclude all other studies, except case-controlled studies.

#### 2.2.2 Type of participants

In this study, the reports of patients with a suspected FRA investigated using PUD will be included.

#### 2.2.3 Type of index test

Index test: PUD will be applied to diagnose participants with FRA. However, the combinations of PUD with other index tests will be excluded.

Reference test: patients with Color Doppler ultrasound-proven FRA will be considered in the control group.

#### 2.2.4 Type of outcome measurements

The outcome measurements consist of sensitivity, specificity, positive likelihood ratio, negative likelihood ratio, and diagnostic odds ratio.

### 2.3 Data sources and search strategy

#### 2.3.1 Electronic searches

The following electronic databases will be searched: MEDLINE, EMBASE, Cochrane Library, Web of Science, Springer, Cumulative Index to Nursing and Allied Health Literature, Allied and Complementary Medicine Database, Chinese Biomedical Literature Database, and China National Knowledge Infrastructure for diagnostic accuracy studies from inceptions to the present without language restrictions. A search strategy for MEDLINE is presented in Table 1. Similar search strategies will also be utilized to other databases.

**Table 1:**
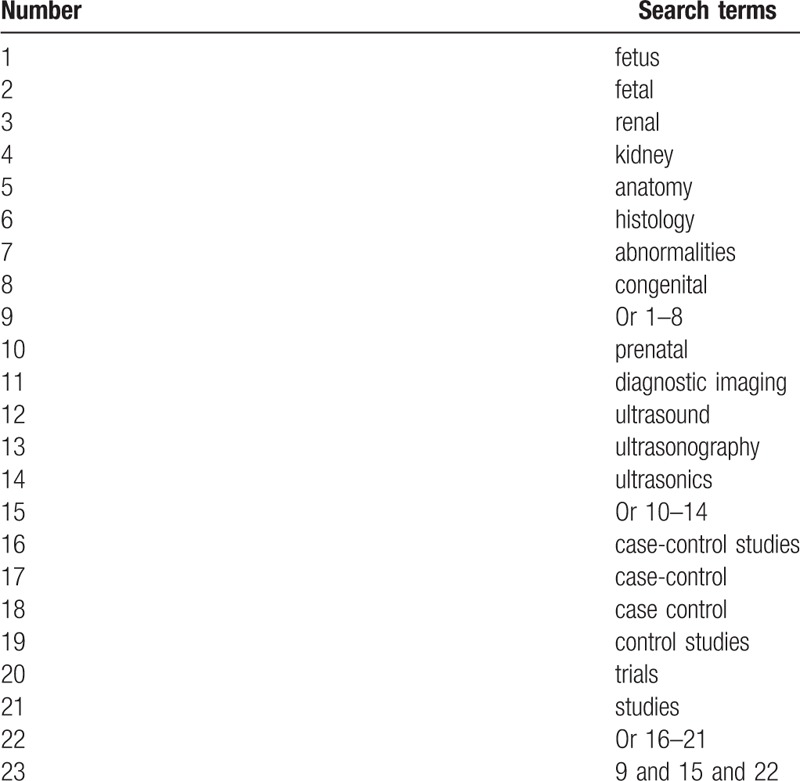
Search strategy builds in MEDLINE.

#### 2.3.2 Other resources

Additional references of included studies and conference proceedings will also be searched.

### 2.4 Data collection and analysis

#### 2.4.1 Selection of studies

Two authors will independently perform titles and abstracts screening procedures, and all unqualified studies will be excluded. We will read full-text of all remaining studies to check their final eligibility criteria if uncertainty will be obtained. Any conflicts between 2 authors will be solved by consensus with the help of a third author. All study selection will be presented in the flowchart.

#### 2.4.2 Data collection and management

A structured data extraction sheet will be built to facilitate the process of data collection from the studies that have been included. Two authors will independently carry out data extraction. Discrepancies between 2 authors will be solved by consensus with a third author if necessary. We will extract following information of title, first author, year of publication, country, study period, study characteristics, patient characteristics, patient selection, eligibility criteria, index test, reference test, and outcome measurements.

### 2.5 Methodological quality assessment

This study will assess the study quality for each entered study using Quality Assessment of Diagnostic Accuracy Studies tool by 2 independent authors.^[21]^ A third author will be invited to solve any divergences between 2 authors regarding the assessment of study quality.

### 2.6 Assessment of heterogeneity

We will measure statistical heterogeneity among included studies using *I*^*2*^ statistic. An *I*^*2*^ value ≤50% is indicative of a low degree of heterogeneity, while An *I*^*2*^ value >50% is indicative of a high degree of heterogeneity.

### 2.7 Statistical analysis

In this study, we will carry out data pooling and statistical analysis using RevMan V.5.3 and Stata V.12.0 software.

#### 2.7.1 Data synthesis

We will pool the data and conduct meta-analysis if heterogeneity is low (*I*^*2*^ ≤ 50%). Otherwise, we will carry out subgroup analysis if the heterogeneity is significant (*I*^2^ > 50%). If there is still very high significant, we will not pool the data, and will perform a bivariate random-effects regression for summary of estimates of sensitivity and specificity.

#### 2.7.2 Subgroup analysis

Subgroup analysis will be conducted on the basis of differences in interventions, comparators, and outcome assessments.

#### 2.7.3 Sensitivity analysis

Sensitivity analysis will be operated by removing low methodological quality studies.

#### 2.7.4 Reporting bias

Reporting bias will be examined using funnel plots if a sufficient number of studies are eligible for inclusion.^[22]^

### 2.8 Ethics and dissemination

This study will use secondary data, thus no ethics approval is needed. Its results are expected to be published in a peer-reviewed journal.

## 3 Discussion

This is the first study to explore the diagnostic accuracy of PUD for FRA in pregnant women. We will comprehensively and systematically search literature sources, as well as the grey record sources to avoid missing more potential studies. The outcomes of sensitivity, specificity, positive likelihood ratio, negative likelihood ratio, and diagnostic odds ratio will be utilized to assess the diagnostic accuracy of PUD for FRA. The findings of this study will summarize up-to-date evidence on the diagnosis of PUD for FRA in pregnant women. It will provide helpful evidence for FRA check and future studies.

## Author contributions

**Conceptualization:** Ying Chen, Wei Liu.

**Data curation:** Ying Chen, Wei Liu.

**Formal analysis:** Ying Chen.

**Investigation:** Ying Chen, Wei Liu.

**Methodology:** Ying Chen.

**Project administration:** Wei Liu.

**Resources:** Ying Chen.

**Software:** Ying Chen, Wei Liu.

**Supervision:** Wei Liu.

**Validation:** Ying Chen, Wei Liu.

**Visualization:** Ying Chen, Wei Liu.

**Writing – original draft:** Ying Chen, Wei Liu.

**Writing – review & editing:** Ying Chen, Wei Liu.
